# A Novel OmpR-Type Response Regulator Controls Multiple Stages of the *Rhizobium etli – Phaseolus vulgaris* N_2_-Fixing Symbiosis

**DOI:** 10.3389/fmicb.2020.615775

**Published:** 2020-12-15

**Authors:** Susana Rodríguez, David Correa-Galeote, Mishael Sánchez-Pérez, Mario Ramírez, Mariel C. Isidra-Arellano, María del Rocío Reyero-Saavedra, David Zamorano-Sánchez, Georgina Hernández, Oswaldo Valdés-López, Lourdes Girard

**Affiliations:** ^1^Programa de Biología de Sistemas y Biología Sintética, Centro de Ciencias Genómicas, Universidad Nacional Autónoma de México, Cuernavaca, Mexico; ^2^Programa de Genómica Computacional, Centro de Ciencias Genómicas, Universidad Nacional Autónoma de México, Cuernavaca, Mexico; ^3^Programa de Genómica Funcional de Eucariontes, Centro de Ciencias Genómicas, Universidad Nacional Autónoma de México, Cuernavaca, Mexico; ^4^Laboratorio de Genómica Funcional de Leguminosas, Facultad de Estudios Superiores Iztacala, Universidad Nacional Autónoma de México, Tlalnepantla de Baz, Mexico

**Keywords:** OmpR-type regulator, nodulation, nitrogen fixation, symbiosis, gene expression, multidrug efflux pumps

## Abstract

OmpR, is one of the best characterized response regulators families, which includes transcriptional regulators with a variety of physiological roles including the control of symbiotic nitrogen fixation (SNF). The *Rhizobium etli* CE3 genome encodes 18 OmpR-type regulators; the function of the majority of these regulators during the SNF in common bean, remains elusive. In this work, we demonstrated that a *R. etli* mutant strain lacking the OmpR-type regulator RetPC57 (ΔRetPC57), formed less nodules when used as inoculum for common bean. Furthermore, we observed reduced expression level of bacterial genes involved in Nod Factors production (*nodA* and *nodB*) and of plant early-nodulation genes (*NSP2*, *NIN*, *NF-YA* and *ENOD40*), in plants inoculated with ΔRetPC57. RetPC57 also contributes to the appropriate expression of genes which products are part of the multidrug efflux pumps family (MDR). Interestingly, nodules elicited by ΔRetPC57 showed increased expression of genes relevant for Carbon/Nitrogen nodule metabolism (*PEPC* and *GOGAT*) and ΔRetPC57 bacteroids showed higher nitrogen fixation activity as well as increased expression of key genes directly involved in SNF (*hfixL, fixKf, fnrN, fixN, nifA* and *nifH*). Taken together, our data show that the previously uncharacterized regulator RetPC57 is a key player in the development of the *R. etli* - *P. vulgaris* symbiosis.

## Introduction

Nitrogen is an essential component of relevant biomolecules (e.g., nucleic acids and proteins) for all living organisms. However, only a few prokaryotic organisms can fix atmospheric nitrogen (reduction of dinitrogen into ammonia) and make it available for bio-assimilation. Within this selected bacterial group are the rhizobia, that fix nitrogen within specialized organs, the nodules, elicited in the roots of several legume species.

The transition from a soil dwelling bacterium lifestyle to forming a symbiotic relationship with a specific partner involves interkingdom chemical-communication, the entrance into an infective stage and eventually a differentiation process to a nitrogen-fixing bacteroid (Murray, 2011; Downie, 2014). This process is tightly regulated by signal molecules that include legume-derived flavonoids and isoflavonoids that attract and are sensed by compatible rhizobia through activating the transcriptional factor NodD (Fischer, 1994). NodD, in turn, activates the transcription of *nodABC* genes that produces signal molecules called Nod Factors (NF) that induce a root-infection process. The NF are chemically decorated lipochitooligosaccharides that are sensed by the legume host through lysine motif receptor-like kinases (LysM) (Oldroyd and Downie, 2008; Oldroyd, 2013). NF perception triggers a series of molecular responses that activate cellular rearrangements enabling rhizobia infection and nodule development (Venkateshwaran et al., 2013). Some of these molecular responses regulate the rhizobia-induced root hair deformations, that are necessary to entrap the rhizobia into an infection chamber and to initiate the infection of the root cortical cells (Fournier et al., 2015; Suzaki et al., 2015). Once in the infection thread rhizobia differentiate into bacteroids and will engage into the nitrogen-fixation process in mature nodules (Spaink, 2000; Oldroyd and Downie, 2008). From the legume host side, a suite of nodulin genes, which finely regulate nodule development and rhizobial infection, are activated (Mylona et al., 1995; Oldroyd et al., 2005; Suzaki et al., 2015). For instance, the activation of the master regulator *Nodule Inception* (*NIN*) is crucial to coordinate both rhizobial and nodule development (Schauser et al., 1999; Liu et al., 2019; Roy et al., 2020). Legumes have also evolved specific molecular mechanism that allow them to select the appropriate symbiotic partner. For instance, in common bean (*Phaseolus vulgaris*), the expression of the subunits *PvNF-YA1*, *PvNF-YB7*, and *PvNF-YC1* of the heterotrimeric Nuclear Factor-Y (NF-Y) transcription factor is important to promote both rhizobial infection and nodule development in common bean but also to select the most efficient *Rhizobium etli* strains to improved nitrogen fixation efficiency (Zanetti et al., 2010; Rípodas et al., 2019).

For an adequate and successful interaction between both symbiotic partners, the multidrug resistance efflux pumps (MDR) have a relevant role protecting bacteria from the plant defense response and the toxic levels of flavonoids (Bhattacharya et al., 2010: Alvarez-Ortega et al., 2013). MDR pumps have the ability to efflux a broad range of compounds such as antibiotics, toxins, antimicrobials and flavonoids, among others (Martinez et al., 2009; Blanco et al., 2016). Bacterial MDR efflux pumps are grouped into five different structural families (Saier and Paulsen, 2001; Li and Nikaido, 2009). In rhizobia, the major facilitator superfamily (MFS) and the resistance-nodulation division (RND) systems contribute to a successful symbiotic nitrogen fixation (SNF) interaction with legumes, processing a variety of symbiotic signals derived from both partners (González-Pasayo and Martínez-Romero, 2000; Lindemann et al., 2010; Eda et al., 2011; Alvarez-Ortega et al., 2013; Santos et al., 2014; Tett et al., 2014). In *Rhizobium etli* (*R. etli*), the absence of the MDR efflux pump RmrAB results in increased sensitivity to flavonoids and decreased ability to nodulate common bean (González-Pasayo and Martínez-Romero, 2000). Other pumps such as BdeAB in *Bradyrhizobium japonicum* positively influence SNF in inoculated soybean (Lindemann et al., 2010). The *Sinorhizobium meliloti* TolC protein is necessary for the secretion of proteins and exopolysaccharide, antimicrobial resistance and for a successful nitrogen-fixing symbiosis (Cosme et al., 2008). Similarly, the SmeAB pump is important in mediating resistance to antimicrobials and in nodulation competitiveness (Eda et al., 2011). In *R. leguminosarum* bv. *viciae* 3841, absence of the SalRAB pump results in increased sensitivity toward salicylic acid but has no effect on nodulation or nitrogen fixation (Tett et al., 2014).

Functional nodules provide the bacteroids an appropriate environment for nitrogen fixation, supplying a carbon source (e.g., malate) and mineral nutrients (e.g., iron and phosphate), as well as the low-oxygen levels required for nitrogen reduction by the oxygen-labile nitrogenase enzyme complex, but sufficient for bacteroid respiration to energize the process (Oldroyd and Downie, 2008; revised in Rutten and Poole, 2019). The low-oxygen environment established in the nodule, differs from that present in other plant structures, and it is the main signal that initiates the activation of nitrogen fixation (*nif*) genes and other bacterial genes involved in maintaining the metabolic adjustments needed to preserve ATP production (*fix* genes) through a series of sophisticated regulatory networks (revised in detail in Fischer, 1994; Dixon and Kahn, 2004; Udvardi and Poole, 2013; Rutten and Poole, 2019, among others).

The molecular basis of SNF and its regulation has been studied in multiple model organisms of the Rhizobiaceae group (Masson-Boivin and Sachs, 2018). These include *R. etli*, a bacterium that establishes a symbiotic relationship with common bean (*Phaseolus vulgaris*), a legume crop with great economic importance worldwide. The genome of *R. etli* strain CE3 is composed of one chromosome and six large plasmids (pRet42a to pRet42f) whose sizes range from 184.4 to 642.5 kb (González et al., 2006). Important advances have been made to elucidate the regulatory mechanisms involved in the symbiotic relationship between *R. etli* and *P. vulgaris*; however, most of this work has focused in just a few signaling modules from the vast repertoire available in the genome of *R. etli*.

The Microbial Signal Transduction Database 3.0 (Gumerov et al., 2020^1^) indicates that the genome of *R. etli* CE3 encodes 677 signaling proteins, which form part of one-component and two-component regulatory systems as well as chemosensory systems and extracytoplasmic sigma factors. These observations strongly suggest that the behavior of *R. etli* is exquisitely controlled by a plethora of signaling systems that respond to a variety of external cues. The two-component systems (TCS) represent one of the most prevalent types of signaling modules in bacteria. The knowledge related to their function, structure, specificity, detection mechanisms and evolution has greatly increased in recent years (for a review, see Casino et al., 2009; Gao and Stock, 2009; Podgornaia and Laub, 2013; Salazar and Laub, 2015). In its most simplified scheme, TCSs are composed of regulatory pairs, consisting of one histidine kinase (HK) and one response regulator (RR) that allow bacteria to deal with different environmental stimuli (Stock et al., 2000). The genome of *R. etli* encodes 47 HKs and 68 RRs (Gumerov et al., 2020; see footnote 1), however, function of most of these remains unknown.

Members of the OmpR/PhoB family of RRs are highly represented among studied genomes and have been described to be involved in metabolism, stress response, virulence, multidrug resistance, and host–microbe interactions among other processes (Barakat et al., 2010; Howden et al., 2011; Delauné et al., 2012; Huang et al., 2013; Ortet et al., 2015; Chakraborty et al., 2017). In *R. etli* CE3, the OmpR family contains 18 RRs. Despite the importance and multiple processes in which OmpR regulators are involved, only two of them have been characterized in *R. etli* CE3. FxkR, the response regulator which controls the microoxic-dependent expression of *fix* genes in *R. etli* (Zamorano-Sánchez et al., 2012) and VirG, the response regulator that activates the expression of *vir* genes involved in type IV pili production (Wang et al., 2017).

Here, we report on the functional analysis of the RHE_PC00057 OmpR-like response regulator (here referred as RetPC57) and demonstrated that it is required for a successful establishment of a symbiotic relationship between *R. etli* and common bean plants. The absence of this regulator resulted in the formation of less nodules and enhanced nitrogen fixation, that were accompanied by changes in the expression of symbiotic genes from both partners. Our results reveal that RetPC57 is a key player in the development of the *R. etli* - *P. vulgaris* symbiosis and further expand our knowledge regarding the contribution of *R. etli* OmpR-type regulators to the control of SNF.

## Results

### *Rhizobium etli* ORF RHE_PC00057 Encodes a Response Regulator Highly Conserved in the *Rhizobium*/*Agrobacterium* Group

**FIGURE 1:**
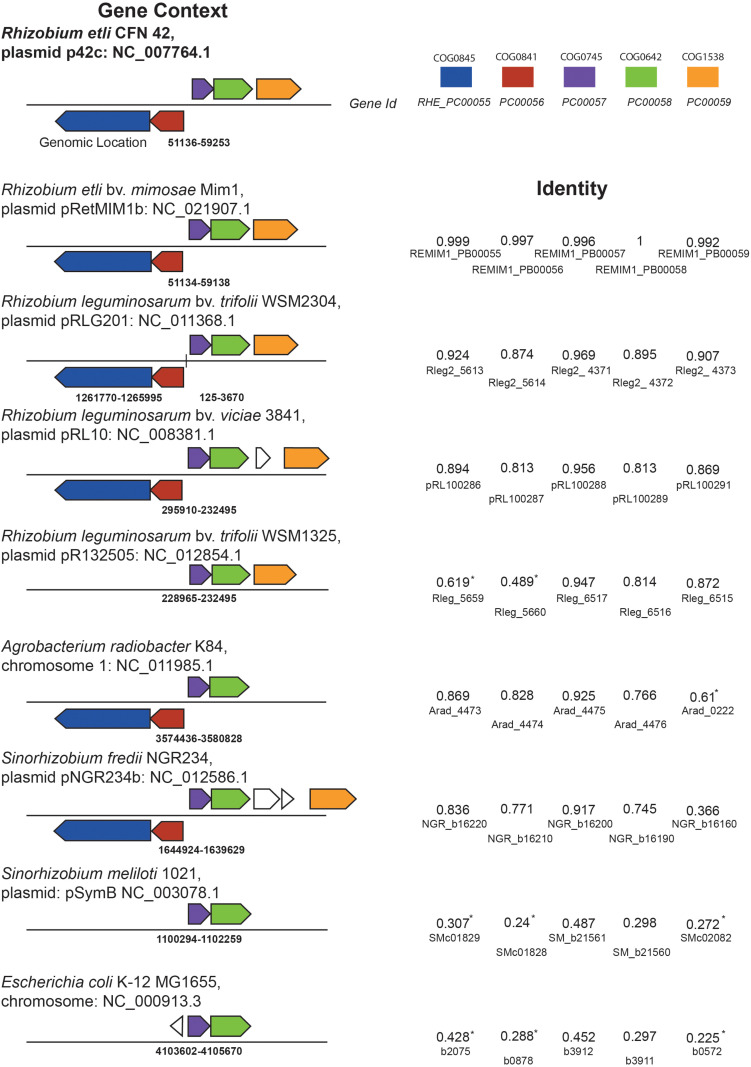
Genomic context analysis of *RetPC57* locus and orthologs genes. Comparative context map of *RHE_PC00055, RHE_PC00056, RHE_PC00057, RHE_PC00058*, and *RHE_PC00059* (*nodTc*) ortholog genes were obtained from IMG tool. Orthologs are shown with matching color. Blue: COG0845, multidrug efflux pump subunit AcrA (membrane-fusion protein). Red: COG0841, multidrug efflux pump subunit AcrB. Purple: COG0745, DNA-binding response regulator, OmpR family, contains REC and winged-helix (wHTH) domain. Green: COG0642, signal transduction histidine kinase. Orange: COG1538, outer membrane protein TolC. Identity based in Sequence Similarity Data Base from KEGG. (*) Indicate genes that are encoded on a distant position in the genome.

**FIGURE 2:**
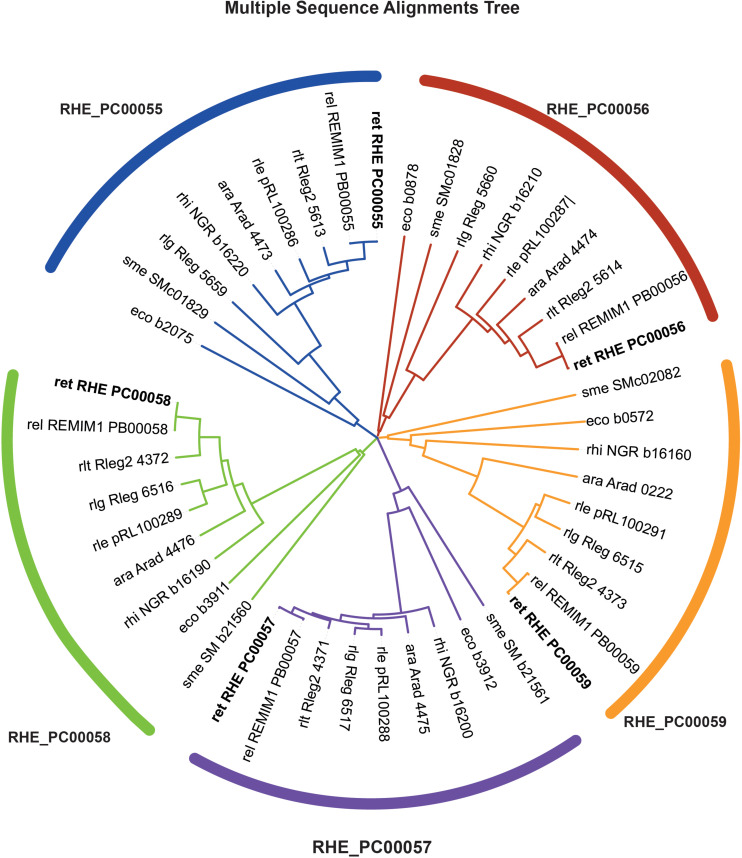
Phylogenetic tree of related genes from *RetPC57* genomic region. A multiple sequence alignment was performed in CLUSTALW and designed on iTOL. *RHE_PC00055* (blue), *RHE_PC00056* (red), *RHE_PC00057* (purple), *RHE_PC00058* (green) and *RHE_PC00059* (*nodTc*, orange) orthologs were obtained from KEGG database (https://www.kegg.jp/). ara, *Agrobacterium radiobacter*; eco, *Escherichia coli* K-12 MG1655; rel, *Rhizobium etli* bv. *mimosae* Mim1; ret, *Rhizobium etli* CFN42; rhi, *Sinorhizobium fredii* NGR234; rle, *Rhizobium leguminosarum* bv. *viciae* 3841; rlg, *R. leguminosarum* bv. *trifolii* WSM1325; rlt, *R. leguminosarum* bv. *trifolii* WSM2304; sme, *Sinorhizobium meliloti* 1021.

From the eighteen OmpR-type regulators included in the MiST 3.0 database, only two have been characterized (Zamorano-Sánchez et al., 2012; Wang et al., 2017). To get further insights about these regulators, we first performed a bioinformatic analysis of all these OmpR-type regulators. The results showed that seven of the *R. etli* CE3 OmpR-like RRs have orthologs with known function in the Rhizobiaceae family; whereas the remaining eleven have less predictable functions. *In silico* analyses revealed that gene *RetPC57*, mapping in megaplasmid pRet42c, encodes for an OmpR-type response regulator with homology (48.7%) to the *Escherichia coli* CpxR regulator. CpxR response regulator form a TCS with the histidine kinase CpxA. The CpxRA is present in many bacteria and regulates a large number of genes in response to periplasmic stress. In *E. coli*, CpxP and NlpE, two periplasmic auxiliary signaling proteins are part of the Cpx system (Danese and Silhavy, 1998; Vogt and Raivio, 2012). A multiple sequence alignment (CLUSTAL W) showed that RetPC57 conserves a high percentage of identity (90 to 99.6%) to proteins annotated as CpxR in bacteria belonging to the *Rhizobium*/*Agrobacterium* group whose function has not been described to date (Supplementary Table 1). The genomic context of *R. etli RetPC57* and its homologs in rhizobia strains was analyzed using public databases^2, 3^ (Figure 1). Interestingly, we observed that these genes are organized similarly in most of the rhizobia strains studied. *RetPC57* homologs are located next to a highly conserved sensor histidine kinase protein gene, RHE_PC00058 (here referred as RetPC58). Similar to RetPC57, the proteins with the highest percentage of identity to RetPC58 (74.5 to 100%) belong to bacteria from the rhizobial group, whereas RetPC58 and CpxA from *E. coli* share only 29.8% identity. In the rhizobial strains, we could not identify genes homologous to *cpxP* or *nlpE* (Danese and Silhavy, 1998; Fleischer et al., 2007). A group of genes predicted to encode proteins belonging to multidrug resistance (MDR) and efflux pumps (Li and Nikaido, 2009) flank *RetPC57* and *RetPC58*. RHE_PC00059, encoding NodTc, an outer membrane efflux protein (OEP) of the TolC superfamily, is located downstream of and in the same orientation as *RetPC57* and *RetPC58*. Transcribed in the opposite direction to *RetPC57* are two genes, RHE_PC00056 and RHE_PC00055, that encode proteins belonging to the RND-family efflux pumps. A phylogenetic tree was constructed to estimate the relationships among *RetPC57* and its adjacent genes with their counterparts in the rhizobia strains studied (Figure 2 and Supplementary Table 1). In summary, these *in silico* analyses indicate that all analyzed genes exhibit a closest relationship between *R. etli* and *R. leguminosarum* strains, followed by *Agrobacterium radiobacter* K84 and *Sinorhizobium fredii* NGR234. In contrast, *S. meliloti* and *E. coli* genes were located on a more distant branch (Figure 2).

### RetPC57 and Symbiotic Conditions Are Required for *RetPC57* Locus Expression

Genes flanking *RetPC57* and *RetPC58* (ORFs RHE_PC00055, RHE_PC00056 and RHE_PC00059) are referred here as *RetPC55*, *RetPC56* and *nodTc*, respectively. RetPC57 and RetPC58 coding regions are located in positions pRet42c: 55707 – 56393 and 56396 – 57685, respectively. This observation led us to hypothesize that these two genes are organized in an operon. To test this hypothesis, we evaluated their gene expression in aerobic cultured bacteria, by reverse transcription polymerase chain reaction (RT-PCR) using total RNA from the wild type strain (WT). The synthesis of cDNA products corresponding to each gene and a single cDNA product corresponding to *RetPC57- RetPC58* genes supports an operon organization for *RetPC57* and *RetPC58* genes (data not shown). Further proof of this operon arrangement was obtained by generating two overlapping β-glucuronidase (GUS) transcriptional fusions. The pPC57-gus fusion contains the putative promoter region whereas the pPC58-gus fusion carries part of the coding region of both genes and overlaps with the end of the pPC57-gus fusion. Both fusions were mobilized into the *R. etli* wild-type (WT) strain by conjugation and GUS-specific activities were determined from bacteria grown in minimal media (MMY) aerobic cultures. Significant Gus activity was only detected from the strain harboring the fusion pPC57-gus, indicating that there is only one promoter in the region and is located upstream of the *RetPC57* gene (Table 1). Most of the experimental studies and bioinformatics searches have revealed that genes encoding for a putative two component system are located adjacent in the genome (Ulrich and Zhulin, 2009). Thus, based on our expression analysis it is very likely that RetPC58 protein is the cognate histidine kinase of the RetPC57 response regulator. The modest level of expression displayed by the *RetPC57* promoter indicates a low basal transcription level of this gene under the assay condition used (Table 1). A similar level of expression was observed under low oxygen condition or in the stationary growth phase (data not shown). In contrast, an increased level of expression of the pPC57-gus fusion was observed in the strain carrying the pQPC57 plasmid, that contains the wild type *RetPC57* gene under the control of a cumate-inducible promoter (Table 1). The highest level of RetPC57 expression was observed in bacteria grown aerobically with cumate (Q) added to the growth media. These results indicate that RetPC57 is needed to activate its own expression and that the conditions tested are not particularly efficient to promote the activation of this regulator (Table 1). The participation of RetPC57 as a regulator of the expression of their adjacent genes *RetPC56* and *nodTc* was analyzed by determining GUS-specific activity. The upstream regions of each gene were fused to the *uidA* reporter gene in plasmids pPC56-gus and pnodTc-gus. The expression of these fusions was analyzed in both the WT and in a WT derivative strain carrying plasmid pQPC57. As shown in Table 1, *RetPC56* and *nodTc* genes displayed very low expression, unaffected by the presence of plasmid pQPC57. However, the expression of these genes was induced when cumate was added to the growth medium (Table 1). These results confirmed that *RetPC56* and *nodTc* genes are under the control of RetPC57 and its expression depends on *RetPC57* expression

**TABLE 1:** Expression analysis of *RetPC57*, *RetPC58*, *RetPC56*, and *nodTc* genes in different *R. etli* derivatives.

Strain	Fusion genotype	β-glucuronidase specific activity^1^	
		MMY^2^	MMYQ^3^
WT/pPC57-gus	*RetPC57:uidA*	45 ± 8	Nd
WT/pPC57-gus/pQPC57	*RetPC57:uidA*	204 ± 40	793 ± 64
WT/pPC58-gus	*RetPC58:uidA*	2 ± 1	Nd
WT/pPC56-gus	*RetPC56:uidA*	5 ± 1	Nd
WT/pPC56-gus/pQPC57	*RetPC56:uidA*	29 ± 2	190 ± 23
WT/pnodTc-gus	*nodTc:uidA*	5 ± 2	Nd
WT/pnodTc-gus/pQPC57	*nodTc:uidA*	43 ± 10	1099 ± 169
WT/pPC57-gus/pLC290	*RetPC57:uidA*	41 ± 8	42 ± 4

*^1^Values are expressed as nmol min^–1^ mg protein^–1^. Data are the mean of three replicates from two independent experiments ± SD. ^2^Y minimal medium. ^3^Y minimal medium + Cumate at a final concentration of 5 μg/ml. Nd: not determined*

**FIGURE 3:**
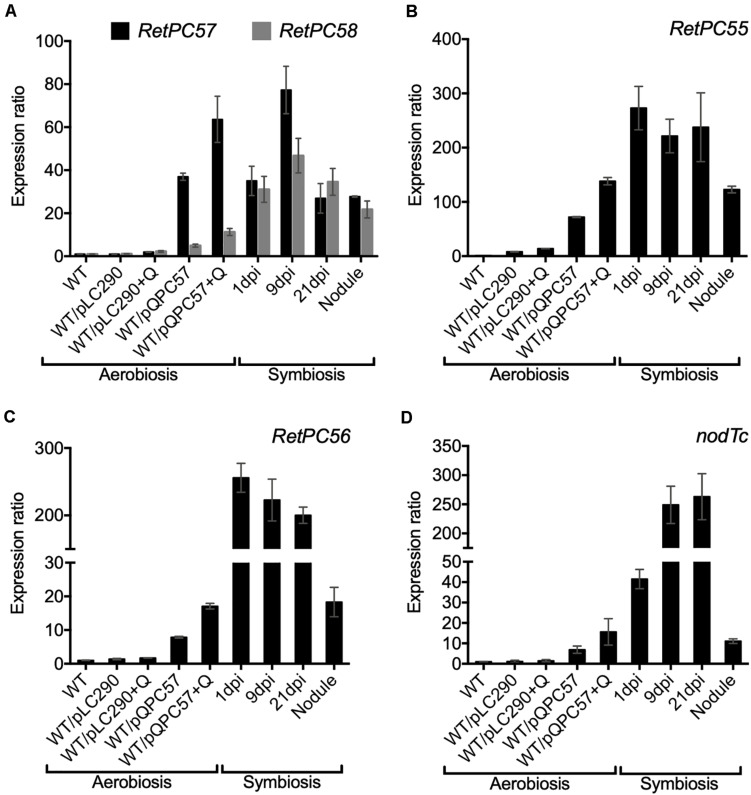
Expression analysis of genes from *RetPC57* genomic region. Expression of **(A)** *RetPC57, RetPC58*, **(B)** *RetPC55*, **(C)** *RetPC56*, and **(D)** *nodTc* genes was determined from aerobic growth cultures of *R. etli* WT, WT/pLC290 and WT/pQPC57 strains, and from common bean roots inoculated with the *R. etli* WT strain, at 1, 9 and 21 dpi and from nodules of 21 dpi. Expression ratios were normalized with the value of WT aerobic condition, set to 1. The *rpoA* was used to normalize gene expression. Data are the mean values of three replicates in a single qRT-PCR experiment. (+ Q) for induction of *RetPC57* gene in strain bearing the pQPC57 plasmid, cumate was added at final concentration of 5 μg/ml.

Next, we performed a transcriptional analysis of the genes from the *R. etli* RetPC57-RetPC58 operon in WT bacteria engaged in symbiosis with common bean plants. The gene expression level was analyzed in roots at 1, 9, and 21 days post inoculation (dpi), from bacteria attached or infecting roots and at 21 dpi from nodules. We observed a similar increase in the level of expression of both genes in symbiotic bacteria and in aerobic cultured bacteria bearing the pQPC57 plasmid (Figure 3A). This result indicates that the symbiotic condition triggers the activation of the *RetPC57* gene. In addition, symbiotic bacteria showed a high expression level of the *RetPC55, RetPC56* and *nodTc* genes, compared to the expression level from bacteria under free-living aerobic conditions (Figures 3B–D). Furthermore, the similarity of the expression profile displayed by these genes with the RetPC57 regulator suggests that the RetPC57 locus is active and functional in *R. etli* symbiosis.

### Absence of RetPC57 Affects the *R. etli* – *Phaseolus vulgaris* Symbiosis

**FIGURE 4:**
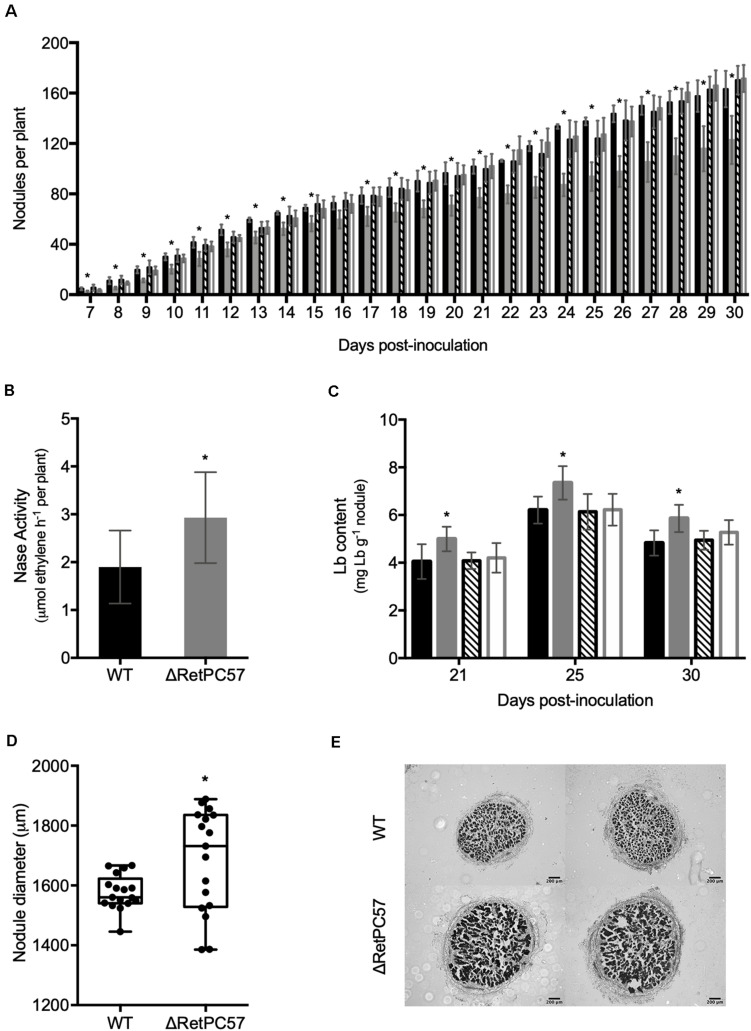
RetPC57 regulator is involved in *R. etli – Phaseolus vulgaris* symbiosis. Common bean seedlings were inoculated with *R. etli* WT (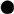), ΔRetPC57 (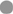), ΔRetPC57/pPC57 (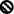), or WT/pFAJ1700 (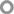) strains and cultivated in growth pouches. **(A)** Inoculated plants were cultivated and the number of nodules formed per plant were determined daily from 7 to 30 dpi. Inoculated plants were cultivated in growth pouches, under controlled conditions to determine: **(B)** Nitrogenase activity (ARA) determined at 21 dpi. **(C)** Leghemoglobin content (Lb) determined in plants harvested after 21, 25, and 30 dpi. **(D)** Diameter from 21 dpi nodules. **(E)** Representative images of light microscopy observations of mature common bean nodule excised at 21 dpi from common bean plants inoculated with WT or ΔRePC57 *R. etli* strains. Transversal sections of nodules from each condition were stained with safranine and visualized by microscopy. Data in **A** and **B**, represent the average from three different experiments, consisting of 6 plants per treatment. Data in **C** represent the average from three biological replicates in three independent experiments. In **D** box plots, horizontal box side represent the first and third quartile while the outside whiskers the minimum and maximum; values represent the average of 20 nodules from different seedlings. (*) significant difference as compared to the values from WT-inoculated roots (*P* ≤ *0.05*, Student’s *t*-test).

To explore the participation of *R. etli* RetPC57 regulator during common bean symbiosis, we constructed a *R. etli* mutant strain with a *RetPC57* gene deleted (ΔRetPC57), and characterized some of its molecular responses of the ΔRetPC57 strain during its symbiotic interaction with common bean. In a first approach, we compared the nodulation kinetics of common bean plants inoculated with either the WT, ΔRetPC57, ΔRetPC57/pPC57 (carrying the WT *RetPC57* gene) or WT/pFAJ1700 (carrying the empty vector) strains. Nodule primordia and young nodules were visible at 7 dpi regardless of the strain used. However, the number of nodules in plants inoculated with the ΔRetPC57 strain was significantly lower compared to that observed in plants inoculated with the WT strain across the different time points tested in this study (Figure 4A). The wild-type phenotype was restored when the ΔRetPC57 mutant was complemented with plasmid pPC57; while the WT/pFAJ1700 showed unaffected nodulation (Figure 4A). The observed nodulation phenotype raises the possibility that the absence of RetPC57 regulator could also affect negatively the nitrogen fixation capacity of the strain. However, an increase in the nitrogenase activity (Figure 4B), a higher leghemoglobin (Lb) content (Figure 4C) and an increase in the size of the nodules (Figures 4D,E), were observed in plants inoculated with the ΔRetPC57 mutant. Taken together, these results suggest that RetPC57 is required for an optimal nodulation, while having a negative impact on nitrogen fixation.

### RetPC57 Is Required for an Appropriate Rhizobial Infection of Common Bean

**FIGURE 5:**
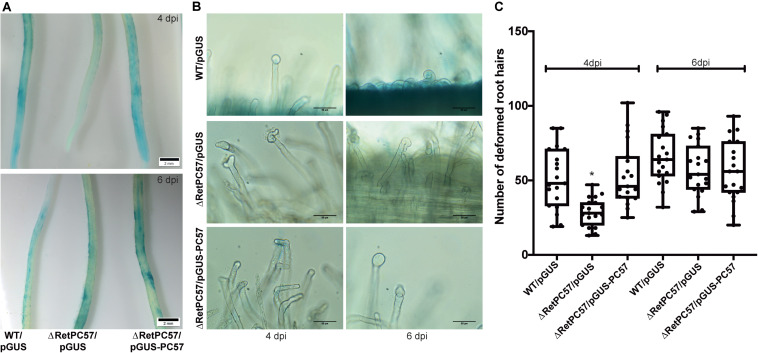
RetPC57 is required for an appropriate rhizobial infection to common bean roots. Roots from common bean seedling were inoculated with *R. etli* WT/pGUS, ΔRetPC57/pGUS, or ΔRetPC57/pGUS-pc57 and were analyzed after 4 and 6 dpi. **(A)** Representative images of GUS-stained common bean roots after rhizobia attachment (bars = 2 mm). **(B)** Representative images of rhizobia-induced root hair deformation (bars = 100 μm). **(C)** Number of rhizobia-induced deformed root hairs. In box plots, horizontal box side represent the first and third quartile while the outside whiskers the minimum and maximum values. Data were obtained from four biological replicates, each one with six roots from different seedlings. (*) significant difference as compared to the values from WT-inoculated roots (*P* ≤ *0.05*, Student’s *t*-test).

The chemotactic response to legume flavonoids and rhizobia attachment to a growing root hair tip, are two crucial steps for the initiation of the rhizobial infection process (Wheatley and Poole, 2018). To further investigate the relevance of RetPC57 in the symbiotic relationship with common bean we explored the attachment capacity to the legume host root hairs of the RetPC57 mutant strain. Common bean seedlings were inoculated with either the WT (WT/pGUS) or the mutant (ΔRetPC57/pGUS) strains, both of them constitutively expressing the *uidA* gene. After 4 dpi, common bean roots inoculated with the WT strain displayed a strong GUS signal in the susceptible zone, whereas only a faint signal was observed in the roots inoculated with the ΔRetPC57 strain (Figure 5A). Interestingly, at 6 dpi we observed no differences in the GUS signal displayed in roots inoculated with either WT or ΔRetPC57 strains (Figure 5A). To further confirm that the delayed attachment of the ΔRetPC57 strain was due to the absence of RetPC57, we performed complementation assays by expressing the *RetPC57* gene under its native promoter (ΔRetPC57/pGUS-pc57). As expected, the common bean roots inoculated with the complemented strain showed a GUS signal similar to that observed in roots inoculated with the WT strain at both 4 and 6 dpi (Figure 5A). These data indicate that RetPC57 is required for the efficient and timely attachment of rhizobia to the common bean roots, the lack of the regulator perhaps delays or affect the efficient communication between rhizobia and its legume host.

Upon attachment to the root hair, rhizobia produce NF, which induce a continuous reorientation of the root hair tip growth, leading to its curling and rhizobia entrapment in the infection chamber (Catoira et al., 2000; Fournier et al., 2015). The fact that RetPC57 seems to regulate rhizobial attachment to the roots prompted us to evaluate whether the absence of this regulator affects the rhizobia-induced root hair deformation. To this end, 2-days-old common bean seedlings were inoculated with either WT (WT/pGUS), mutant (ΔRetPC57/pGUS) or the complemented (ΔRetPC57/pGUS-pc57) strains. After 4 dpi, the roots inoculated with ΔRetPC57 or the WT strain showed similar type of root hair deformation events (Figure 5B). However, a quantitative difference of root hair deformation events was observed; while roots inoculated with the WT strain developed on average 50 rhizobia-induced root hair deformation events, the roots inoculated with the mutant strain developed on average 20 events of deformed root hairs (Figure 5C). Interestingly, after 6 days of inoculation similar numbers of root rhizobia-induced root hair deformation events was observed despite the strain used as inoculant (Figure 5C). The delayed phenotype observed in the infection process of the ΔRetPC57 strain was complemented by the expression of *RetPC57* in *trans* (Figures 5B,C). These data indicate that the absence of RetPC57 results in a delay of the mutual recognition between both symbiotic partners, thus this regulator may play a role in the rhizobia infection process.

### The Absence of RetPC57 Affects the Expression of Both Rhizobial and Plant Symbiotic-Genes Involved in the Rhizobial Infection Process

**FIGURE 6:**
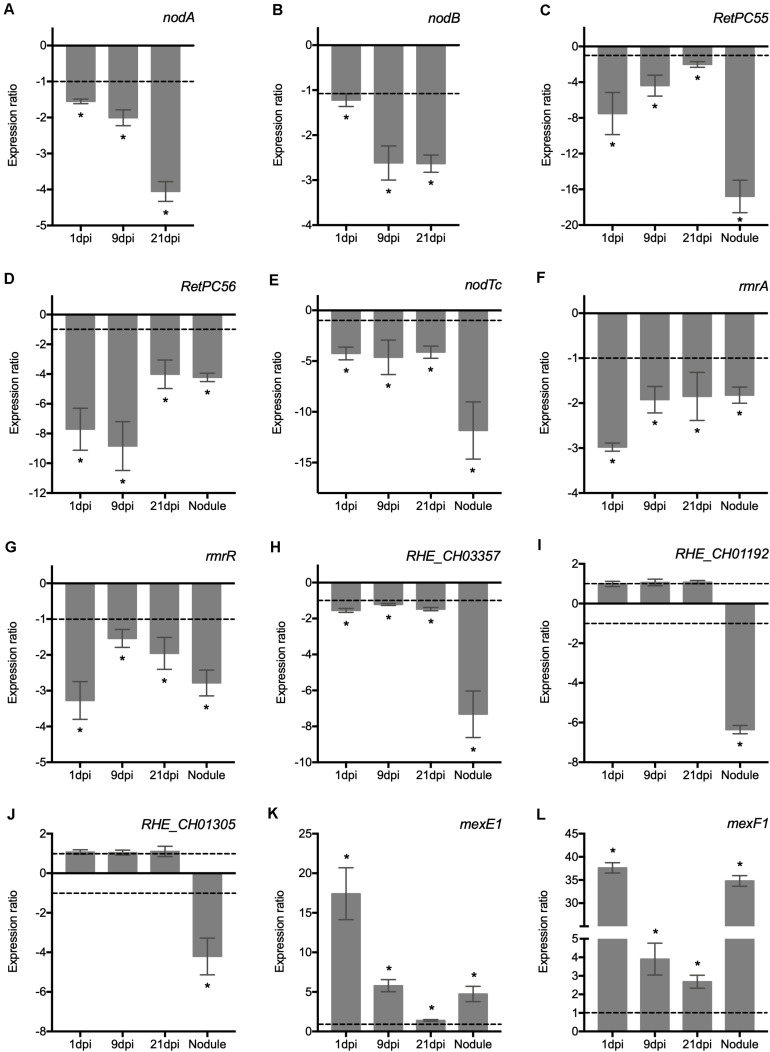
RetPC57 is required for the adequate expression of genes related to nodulation factor synthesis and multidrug resistance efflux pumps in common bean roots. Common bean seedlings were inoculated with WT and ΔRetPC57 *R. etli* strains. Normalized expression ratios (ΔRetPC57: WT) of the indicated rhizobia **(A–L)** genes, were determined from 1, 9, and 21 dpi roots and 21 dpi nodules. The *rpoA* was used to normalize gene expression of bacterial and plant genes, respectively. When ratio values were lower than 1, the inverse value was estimated and the sign was changed. Data are the mean values of three replicates in three independent samples in a single qRT-PCR experiment. (*) significant difference as compared to the values from WT-inoculated plants (*P* ≤ *0.05*, Student’s *t*-test).

Secondary metabolites produced by plant roots are secreted to the rhizosphere and play important roles in the interaction between plants and soil microbes. Flavonoids secreted by the host plant roots activate in rhizobia the expression of nodulation genes necessary for the synthesis and secretion of NFs. To establish a successful infection of legume roots by rhizobia, both the biological activity of NF and the physiological functions of the multidrug resistance (MDR) efflux systems are essential (González-Pasayo and Martínez-Romero, 2000; Bhattacharya et al., 2010; Lindemann et al., 2010). In order to gain insight into the potential role of RetPC57 as transcriptional activator and/or repressor, we conducted an expression analysis of the genes related to rhizobial infection and early nodulation in plants inoculated with the WT or the ΔRetPC57 strain after different post inoculation times points (Figure 6). The absence of RetPC57 had a negative impact on the abundance of the transcripts of the bacterial NF-synthesis genes *nodA* (Figure 6A) and *nodB* (Figure 6B). It has been reported that expression of the MDR systems is subject to multiple levels of regulation that includes a regulator member of the TetR transcriptional repressors and a global transcriptional regulator such as the TCS. In *E. coli*, the TCS EvgAS, PhoPQ and BaeSR participate in regulating/modulating the expression of these transporters (Ramos et al., 2005; Li and Nikaido, 2009). Thus, we investigated whether the expression of different *R. etli* genes predicted to encode putative proteins members of the MDR efflux systems is influenced by the absence of the RetPC57 regulator. Our previous expression analysis of *RetPC55, RetPC56* and *nodTc* genes performed in the WT under both aerobic and symbiotic conditions suggests that these genes are under the control of RetPC57 regulator (Table 1 and Figures 3B–D). If that is the case, the expression of these genes in plants inoculated with the ΔRetPC57 should be negatively affected. As shown in Figures 6C–E, the level of expression of *RetPC55, RetPC56* and *nodTc* genes was reduced in comparison with the expression observed in plants inoculated with the WT strain. These results indicate that RetPC57 is needed as a positive regulator for its expression. We also analyzed the expression of other genes that are predicted encode for both proteins belonging to the RND-family efflux pumps (*mexF1*-*mexE1* and *RHE_*CH01305) and proteins belonging to the MFS-type multicomponent efflux systems (*rmrA, RHE_CH01192* and *RHE_CH03357*) (See Supplementary Table 2 for additional information of these genes and their orthologs). Similar to *nodAB*, *RetPC55, RetPC56* and *nodTc*, the expression of *rmrA* and *RHE_CH03357* genes, as well as the expression of *rmrR*, the putative regulator of *rmrAB* genes, were significantly reduced in plants inoculated with the ΔRetPC57 mutant compared to those inoculated with the WT strain (Figures 6F–H). The expression of *RHE_CH01192* and *RHE_CH01305* genes was negatively affected only in 21 days-old nodules elicited in plants inoculated with the ΔRetPC57 strain, and not in the roots at 1, 9 or 21 dpi (Figures 6I,J). In contrast, the deletion of *RetPC57* resulted in a significant induction of the expression of genes *mexE1* and *mexF1* (Figures 6K,L). These results indicate that in *R. etli*, the RetPC57 regulator is important for the proper expression of genes associated with the production of the nodulation factor and genes associated with the transport of chemical signals during the establishment of an effective *R. etli – P. vulgaris* symbiosis.

**FIGURE 7:**
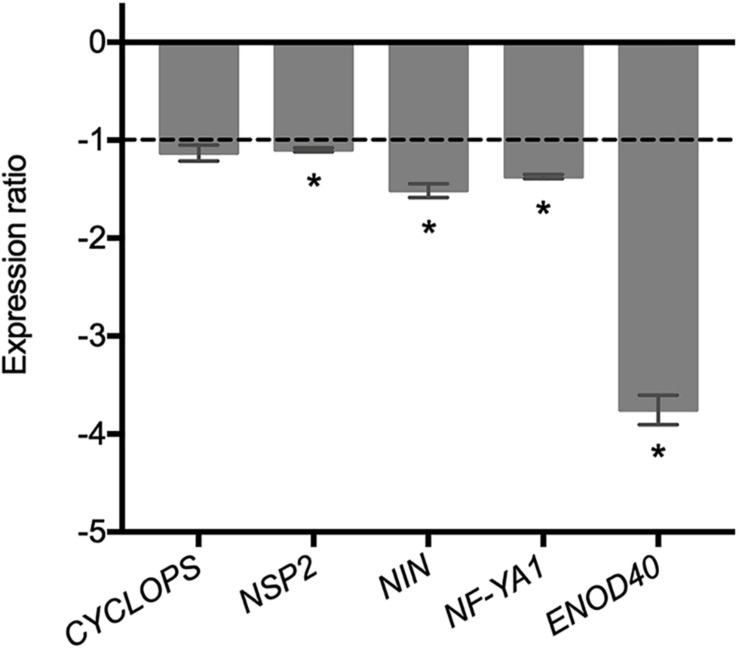
Expression of common bean genes related to rhizobial infection and early nodulation in common bean roots inoculated with the RetPC57 mutant. Common bean seedlings were inoculated with WT and ΔRetPC57 *R. etli* strains. Normalized expression ratios (ΔRetPC57: WT) of the indicated common bean genes were determined from 1 dpi roots. The ubiquitin *UBC9* was used to normalize gene expression. When ratio values were lower than 1, the inverse value was estimated and the sign was changed. Data are the mean values of three replicates in three independent samples in a single qRT-PCR experiment. (*) significant difference as compared to the values from WT-inoculated plants (*P* ≤ *0.05*, Student’s *t*-test).

The fact that the expression of rhizobial genes involved in the mutual recognition of the legume host is affected in the ΔRetPC57 mutant prompted us to assess the expression of the plant genes CYCLOPS, NSP2, NIN, and NF-YA involved in the rhizobial infection process (Oldroyd, 2013; Nova-Franco et al., 2015; Roy et al., 2020) in common bean roots inoculated with the ΔRetPC57 mutant in comparison to plants inoculated with the WT strain after 1 dpi. We found that except for CYCLOPS, the expression of the common bean symbiotic-related genes, was lower in roots inoculated with the ΔRetPC57 strain (Figure 7). Additionally, our expression analysis showed a reduction of the expression of the common bean ENOD40 nodulin gene, involved in nodule development (Kumagai et al., 2006; Oldroyd and Downie, 2008), in roots inoculated with the ΔRetPC57 strain (Figure 7). Altogether, our expression data further support the notion that the presence of the regulator RetPC57 is necessary for the adequate expression of both bacterial and plant genes that are determinant during the early stages of the rhizobia-legume symbiosis.

### Increased Expression of Essential Symbiotic Genes in Bacteroids and Nodules Elicited by the ΔRetPC57 Mutant Strain

**FIGURE 8:**
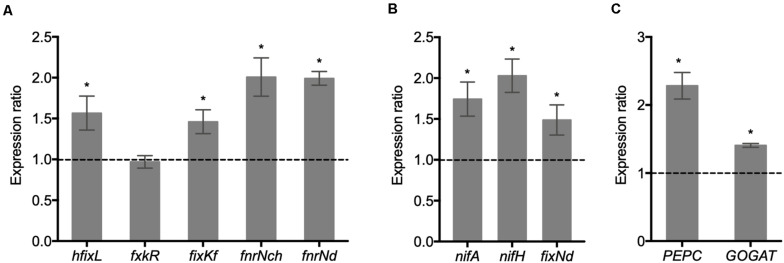
Expression of essential symbiotic genes in nodules elicited by the WT or ΔRetPC57 strains Common bean seedlings were inoculated with *R. etli* WT or ΔRetPC57 strains. Normalized expression ratios (ΔRetPC57: WT) from the indicated rhizobia **(A,B)** or common bean **(C)** genes were determined from nodules 21 dpi. The *rpoA* and the ubiquitin *UBC9* were used to normalize gene expression of bacterial and plant genes, respectively. Data are the mean values of three replicates in three independent samples in a single qRT-PCR experiment. (*) significant difference as compared to the values from WT-inoculated plants (*P* ≤ *0.05*, Student’s *t*-test).

The regulatory mechanism controlling SNF involves the participation of transcriptional factors that control the expression of rhizobia *nif* and *fix* genes. Because the inoculation of common bean plants with the ΔRetPC57 mutant improves its nitrogen fixation capabilities (Figures 4B,C), we explored the expression of *hfixL*, *fxkR*, *fixK*f, *fnrN*ch and *fnrN*d genes in bacteroids from nodules of plants inoculated with either the WT strain or the ΔRetPC57 mutant strain. Our analysis revealed that, except for *fxkR*, the expression of these genes was higher in plants inoculated with the ΔRetPC57 mutant compared with plants inoculated with the WT strain (Figure 8A). We also evaluated the expression of *nifA*, *nifH and fixN*d, which are indispensable for nitrogen fixation. The absence of RetPC57 resulted in an increase in the expression of these genes (Figure 8B). As shown in Figure 8C, the increased expression of bacterial *nif* and *fix* genes in nodules from plants inoculated with the ΔRetPC57 mutant strain correlated with a higher expression of genes coding for relevant carbon and nitrogen metabolism enzymes in nodules, such as phosphoenolpyruvate carboxylase *(PEPC)* and glutamate synthase (*GOGAT)* genes, respectively. Altogether, these data suggest that the negative regulation of nitrogen fixation exerted by RetPC57, involves changes in the expression of both bacterial and nodule genes implicated in nitrogen fixation and in carbon and nitrogen metabolism inside the nodule.

## Discussion

This study provides new insights regarding the involvement of the OmpR-type response regulators of *R. etli* CE3 in different stages/processes of its symbiosis with common bean plants. Preceding work has shown the participation of a variety of OmpR-type regulators in the control of SNF (Lipa and Janczarek, 2020). For instance, in *S. meliloti* ChvI and FeuP are important for alfalfa root invasion through regulating the production of succinoglycan and cyclic glucans, respectively (Griffitts et al., 2008; Bélanger et al., 2009; Wang et al., 2010; Vanderlinde and Yost, 2012). PhoB regulates the response to phosphate limitation in *S. meliloti* and induces the expression of genes involved in phosphorus uptake, metabolism as well as exopolysaccharide production and cell protection genes (Bardin and Finan, 1998; Mendrygal and González, 2000; Krol and Becker, 2004; Yuan et al., 2006). In phosphate limitation, PhoB positively regulates the expression of genes involved in the production of galactoglucan EPSII required for elongation of the infection thread in the legume symbiosis and for protection against plant defense responses (Niehaus et al., 1993; Fraysse et al., 2003; Bahlawane et al., 2008). On the other hand, Rem regulates flagellar gene expression in *S. meliloti* and *R. leguminosarum* biovar *viciae*; its absence affects the ability of these bacteria to swim and perhaps its attachment to biotic surfaces such as legume roots (Rotter et al., 2006; Tambalo et al., 2010). The cell cycle regulator CtrA controls the expression of genes important for *S. meliloti* saprophytic and symbiotic life styles (Barnett et al., 2001; De Nisco et al., 2014; Xue and Biondi, 2019). FxkR regulators, from *R. etli* CE3 and *S. meliloti* SM11, regulate the expression of *nif* and *fix* genes required for the nitrogenase activity and for microaerobic respiration inside of nodules of common bean and alfalfa, respectively (Zamorano-Sánchez et al., 2012; Reyes-González et al., 2016).

In this work, we showed that RetPC57, a previously uncharacterized response regulator from the OmpR family, plays a major role in the symbiosis between *R. etli* and common bean. Genomic context analysis reveals that both, the *RetPC57* – *RetPC58* operon and the *RetPC55*, *RetPC56* and *nodTc* genes are highly conserved and maintain a similar genetic organization in nitrogen-fixing bacteria. Such conservation suggests that this group of genes co-evolved and possibly conserve a similar function. The structural organization of the efflux pumps of the RND family comprises a transporter protein located in the inner membrane, a membrane fusion protein located in the periplasmic space and an outer membrane protein located in the outer membrane of the bacteria (Piddock, 2006). Our data indicate that *RetPC55*, *RetPC56* and *nodTc* encode for this type of proteins that may comprise the structural components of an efflux pump, whose expression is under the control of RetPC57 in response to plant exudates and that contributes to the successful interaction of *R. etli* with common bean. This proposed efflux pump may participate, together with other components such as NodI and NodJ, in the secretion of the NFs and additional rhizobial-derived signal molecules such as succinoglycans which play a crucial role in the progression of the rhizobial infection beyond the root epidermal cells (Kawaharada et al., 2015; Maillet et al., 2020). In *R. etli* and in *R. leguminosarum* bv. *trifolli* mutations in *nodI* and *nodJ* genes affect the amounts of NFs secreted (McKay and Djordjevic, 1993; Cárdenas et al., 1996). The *R. etli* NodI and NodJ mutants presented a delayed nodulation phenotype and a reduction in the number of nodules formed suggesting that additional transport systems are involved in the secretion of NF in *R. etli* (Cárdenas et al., 1996). Functional characterization of *RetPC55* and *RetPC56*, i.e., through phenotypical analysis of mutants deleted in these genes is needed to vulnerate this hypothesis.

Multidrug resistance efflux systems have shown to contribute to a successful symbiotic interaction between nitrogen-fixing bacteria and their host plants as they prevent accumulation of plant-derived toxic compounds (González-Pasayo and Martínez-Romero, 2000; Karunakaran et al., 2009; Lindemann et al., 2010; Eda et al., 2011; Santos et al., 2014; Tett et al., 2014). The genome of *R. etli* CFN42 possesses 44 genes encoding putative antimicrobial efflux pumps (Ormeño-Orrillo et al., 2012). In this study, we have demonstrated that the functionality of the RetPC57 regulator affects the symbiotic transcriptional response of *R. etli* genes that are part of the MDR efflux systems. Previously it has been shown that *R. etli* RmrAB mutants are affected in nodulation with bean plants and are more sensitive to phytoalexins, flavonoids and salicylic acid (González-Pasayo and Martínez-Romero, 2000). Interestingly, in *R. leguminosarum* bv. *viciae* 3841 a mutation in *rmrA* does not affect the antimicrobials susceptibility of the strain. Not only the expression of *rmrA* gene had been found to be different between these two closely related bacteria, contrary to what is observed in *R. leguminosarum* bv. *viciae* 3841 (Tett et al., 2014), we found that in *R. etli* the *RHE_CH1192* gene (the putative ortholog of the Rlv3841*salA* gene) was expressed inside the nodules in the presence of a functional RetPC57. We also observed that *mexE1* and *mexF1* genes are induced in the RetPC57 mutant. In *R. etli* the role of these genes is unknown, however, in *A. tumefaciens* mutants defective in these genes are less virulent in response to acetosyringone and more sensitive to the toxic effects of chloramphenicol (Binns and Zhao, 2020). Previously, the expression of the *R. etli nodTc* gene was reported as constitutive and its mutation does not affected nodulation with common beans nor sensitivity to toxic compounds (Hernández-Mendoza et al., 2007). However, in this work we have shown that the expression of the *nodTc* gene was induced in symbiosis. The discrepancy between Hernández-Mendoza et al. (2007) and this work could be due to the presence of multiple efflux pumps with redundant functions. In addition, the host-specificity of efflux pumps functions has been demonstrated (Lindemann et al., 2010). For example, our results clearly shown that *RetPC55*, *RetPC56* and *nodTc* genes were induced in symbiosis with common bean plants. Conversely transcriptomic analysis of *R. leguminosarum*, bacteroids reported that pRL100286-87 and pRL100291 genes (the putative orthologs of these genes) were down-regulated in bacteroids of common bean and pea nodules (Green et al., 2019). Even more, mutants in pRL100286-87 genes improved bacterial colonization in pea roots (Wheatley et al., 2020). In *R. leguminosarum* bv. *viciae* mutants in the *nodT* gene led to a delay in nodulation on *Trifolium subterraneum* but not on *V. sativa* plants (Canter Cremers et al., 1989). However, in *R. leguminosarum* bv. *trifolli* nodulation is not affected in a NodT mutant (Spaink et al., 1995).

The specific mechanism of action of the RetPC57 regulator is yet unclear. One possibility is that the RetPC57-RetPC58 TCS promotes, directly or indirectly, the adequate expression of the *nod* genes required for the optimal synthesis and secretion of the NFs indispensable for the infection and nodule initiation. The participation of some TCS had been reported as regulators of the expression of the MDR systems, thus another possibility is that this TCS may contribute to prevent the accumulation of toxic plant compounds to protect the bacteroid in the nodules from toxic levels of flavonoids and toxic plant compounds through modifying the expression of the MDR systems. Future experimental evidence is needed to explore these possibilities.

In this work we showed that a ΔRetPC57 mutant strain used as common bean inoculant presented a delayed attachment to roots and formed less nodules, but it showed higher nitrogenase activity in comparison to the WT-inoculated common bean plants. The optimal communication between rhizobia and its host plant ensures the activation of two plant genetic programs that independently control the rhizobial infection and the nodule developmental (Tsyganov et al., 2002; Suzaki et al., 2015; Liu et al., 2019; Roy et al., 2020). The reduced number of nodules elicited by the ΔRetPC57 mutant could be, at least partially, explained by the reduced expression of genes involved in the production of the NF (i.e., *nodA* and *nodB*). Furthermore, plants inoculated with the ΔRetPC57 strain showed reduced expression of genes participating in the plant genetic programs controlling the rhizobial infection process (i.e., *CYCLOPS*, *NSP2*, *NIN* and *NF-YA*) and nodule development (i.e., *ENOD40*), compared with plants inoculated with the WT strain. Different studies in several legumes indicate that a delay in the activation of the rhizobial infection process results in a delay in the activation of the nodule developmental program (Oldroyd and Downie, 2008; Murray, 2011; Oldroyd et al., 2011; Roy et al., 2020). In agreement, the delay observed during initial infection by the ΔRetPC57 strain appears to be translated into a decreased ability to promote nodulation.

Nitrogen fixation activity was higher in nodules occupied by the strain lacking RetPC57; this is in line with an increased expression of bacterial genes involved in microaerobic respiration (*hfixL, fixKf, fnrNch, fnrNd and fixN*) and in nitrogenase biosynthesis (*nifA* and *nifH*). It is still unclear if RetPC57 exerts a direct or an indirect regulation on these genes. *fixKf* is the only gene known to be regulated by an OmpR-like protein (FxkR) (Zamorano-Sánchez et al., 2012) and it is not known if RetPC57 could recognize the same *cis*-regulatory site as that recognized by FxkR. It is possible that RetPC57 activates additional transcriptional regulators that, in turn, promote the expression of symbiotic genes. In this direction, experiments to understand the global gene expression exerted by the RetPC57 regulator are underway in our laboratory.

Not only bacteroid genes are affected by the absence of RetPC57, but plant nodule genes that encode the phosphoenolpyruvate carboxylase (PEPC) and the glutamate synthase (GOGAT), crucial for an efficient carbon and nitrogen metabolism in the nodule (Udvardi and Poole, 2013) also showed increased expression in ΔRetPC57-elicited nodules. This altered common bean genes expression likely responds to changes in the requirement of carbon by the bacteroids and/or to the excess of ammonia from enhanced nitrogen fixation. It is largely known that nitrogen fixation in the root nodules is an energetically expensive process, thus legume plants control the number of nodules formed mainly through the regulatory mechanism called autoregulation of nodulation (AON) (Reid et al., 2011). Here we show that nodules elicited by the ΔRetPC57 mutant are effective, these are even bigger and with an increased capacity to fix nitrogen than those elicited by WT. The later indicates that the functionality of the ΔRetPC57 bacteroids compensate the less number of nodules elicited.

## Conclusion

In conclusion, our results unveiled a previously uncharacterized OmpR-like regulator, RetPC57, that adds a new element to the sophisticated transcriptional regulatory circuits that govern symbiosis between *R. etli* and common bean plants.

## Materials and Methods

### Bacterial Strains, Plasmids, and Growth Conditions

The bacterial strains and plasmids used in this study are listed in Supplementary Table 3. *E. coli* strains were grown at 37°C in Luria-Bertani (LB) medium supplemented with the appropriate antibiotics. *Rhizobium* strains were grown at 30°C in peptone-yeast (PY) medium supplemented with CaCl_2_ (7 mM) (Noel et al., 1984) or in Y minimal medium (MMY) with succinate (10 mM) and ammonium chloride (10 mM) as carbon and nitrogen sources, respectively (Bravo and Mora, 1988). Antibiotics were used at the following concentrations: carbenicillin (Cb), 100 μg ml^–1^ (*E. coli*); fosfomycin (Fm),100 μg ml^–1^ (*Rhizobium*); gentamicin (Gm), 15 μg ml^–1^ (*E. coli* and *Rhizobium*); kanamycin (Km), 30 μg ml^–1^ (*E. coli* and *Rhizobium*); nalidixic acid (Nal), 20 μg ml^–1^ (*Rhizobium*); streptomycin (Sm), 200 μg ml^–1^ (*Rhizobium*); tetracycline (Tc), 10 μg ml^–1^ (*E. coli* and *Rhizobium*). β-galactosidase activity was routinely used for selection of recombinant plasmids. 5-bromo-4-chloro-3-indolyl-β-D-galactoside (X-gal) was used in plates at 20 μg ml^–1^. When required, sucrose was added at 12% (W/V). For growth conditions, cultures were grown to mid-exponential phase in PY medium. Cells were collected by centrifugation, washed with sterile MMY, and concentrated 100-fold. MMY were inoculated with these suspensions at an initial optical density at 540 nm (O.D._540_) of 0.05. Cultures were grown with shaking (200 rpm) for 6 h at 30°C. For stationary growth phase, cultures were grown for 12 h at 30°C with shaking (200 rpm). For microaerobic growth conditions, cultures were prepared as described in Girard et al. (1996). For cumate (Q) induction experiments cells were cultivated with cumate at a final concentration of 5 μg/ml.

### Microbiological and Recombinant DNA Methods

Genomic and plasmid DNA isolation, digestion with restriction enzymes, ligations, agarose gel electrophoresis and *E. coli* transformation were performed using standard protocols (Sambrook et al., 1989). Enzymes used for DNA restriction and modification were purchased from Thermo Scientific^TM^ and used according to the manufacturer’s instructions. Conjugative transfer of plasmids from *E. coli* to *R. etli* was done by triparental crosses using pRK2013 as conjugation helper. For determination of plasmid profiles, a modified Eckhardt procedure was used (Hynes and McGregor, 1990).

### PCR Amplification

Specific PCR primers were designed using the Oligo 7 software and were synthetized at the Unidad de Síntesis Química IBt-UNAM. PCR amplifications were done in a Veriti 96 well Thermal Cycler (Applied Biosystems). High Fidelity Pol DNA Polymerase (Jena Bioscience) was used in PCR reactions following the manufacturer’s instructions. Cycling regime was determined according the primers melting temperature and the length of the DNA fragment to be amplified. Supplementary Table 4 shows the primer sequences used in this work.

### Plasmid Construction

To construct a plasmid useful for homogenotization lacking the *RetPC57* gene, the two regions flanking the gene were amplified with specific primers (Supplementary Table 4). The two resulting PCRs products were then fused using the overlapping extension PCR methodology (Shevchuk et al., 2004). The assembled product was then recovery using the proper primers and cloned into the pJET1.2/blunt cloning vector (Thermo Scientific^TM^) and sequenced to confirm that the fused product was correctly obtained with no nucleotide changes. The appropriate restriction fragments were subcloned into the suicide plasmid pK^∗^*mobsacB* (Schäfer et al., 1994) yielding plasmid pK:Δpc57.

To generate plasmids for expression analysis, regulatory regions of the desired genes were cloned in the broad-host plasmid (Girard et al., 2000) carrying a promoterless *uidA* gene. The intergenic region *RetPC57* and *RetPC56* was obtained by PCR using primers Up-PC57gus and Lw-PC57gus. The 928 bp *Eco*RI fragment was cloned in both directions in pBBMCS53 to obtain plasmids pPC57-gus and pPC56-gus. The correct orientation of the fragments in plasmids was verified with primer gusLw and the corresponding upper primer of each gene. The transcriptional fusion *RetPC58:uidA* was constructed using primers Up-PC57gus and Lw-PC58gus. The 1393 bp PCR product was cloned into the pCR2.1 TOPO (Invitrogen) vector. Then, a 528 bp *Eco*RI-*Xho*I fragment was clone in pBBMCS53 plasmid to obtain plasmid pPC58-gus. Plasmid pnodTc-gus carrying *nodTc:uidA* transcriptional fusion was constructed by cloning the 1210 bp *Sac*I-*Sal*I fragment synthesized by PCR using primers Up-nodTcgus and Lw-nodTcgus.

A plasmid carrying the *R. etli RetPC57* gene expressed from the cumate-inducible promotor (P_*R/cmtO*_) was obtained by cloning the *RetPC57* coding region in the broad-host range vector pLC290 (Chubiz et al., 2013). To that end, primers Up-QPC57 and Lw-QPC57 were used to amplify a product of 711 bp by PCR. The amplification product was cloned into the pJET1.2/blunt (Thermo Scientific^TM^) vector and subsequently sequenced. The insert was then subcloned into the pLC290 plasmid with the *Xba*I-*Spe*I sites introduced by the primers, to obtain pQPC57 plasmid.

To construct plasmids carrying the *RetPC57* gene expressed from its native promoter, a 1,095 bp product was obtained by PCR, using total DNA from strain CE3 and the specific primers Up-PC57-Cpl and Lw-PC57-Cpl. The amplification product was cloned into the pJET1.2/blunt (Thermo Scientific^TM^) vector and subsequently sequenced. Then, the *Nsi*I*-Xho*I fragment was subcloned into the conjugative plasmids pFAJ1700 (Dombrecht et al., 2001) and pGUS (pFAJ1700/p*lacZ*:*uidA*, Isidra-Arellano et al., 2018) to generate the plasmids pPC57 and pGUS-pc57, respectively.

### *Rhizobium etli* Mutant Derivatives

Replacement of the *R. etli RetPC57* wild-type allele by the respective deleted mutant allele in the plasmid pK:Δpc57, was carried out by homogenotization using the *sacB* marker present in the donor plasmid. Double recombinants were selected as Sac^*R*^, Sm^*R*^, Km^*S*^ transconjugants. Eckhardt type gels were used to verify the absence of genomic rearrangements (Hynes and McGregor, 1990). To confirm the lack of the gene, PCR reactions using genomic DNA from both WT and candidates were done using the specific internal primers Up-RetPC57 and Lw-RetPC57.

*Rhizobium etli* WT/pLC290, WT/pQPC57, WT/pPC57-gus, WT/pPC56-gus, WT/pPC58-gus, WT/pnodTc-gus, WT/pPC57-gus/pLC290, WT/pPC57-gus/pQPC57, WT/pPC56-gus/pQPC57, WT/pnodTc-gus/pQPC57, WT/pFAJ1700, WT/pGUS, ΔRetPC57/pGUS ΔRetPC57/pPC57, and ΔRetPC57/pGUS-pc57 strains were obtained by triparental mating using *E. coli* DH5a strain carrying either plasmids pLC290, pQPC57, pPC57-gus, pPC56-gus, pPC58-gus, pnodTc-gus, pFAJ1700, pGUS, pPC57 or pGUS-pc57 and selected with the appropriate antibiotics (See Supplementary Table 3). Plasmid profiles were analyzed by Eckhardt.

### Measurement of GUS Activity

Aerobic cultures of *R. etli* strains harboring transcriptional fusions were grown as described previously (Girard et al., 2000). Quantitative GUS activity was determined on 3.0 ml cultures samples using 4-nitrophenyl β-D-glucuronide as substrate, as described previously (Girard et al., 2000). Data were normalized to total cell protein concentration using Pierce^TM^ BCA Protein Assay Kit (Thermo Scientific) over a second set of 3.0 ml samples. Specific activities are reported in nanomoles of product per minute per milligram of protein.

### Plant Material and Growth Conditions

Common bean *Phaseolus vulgaris* cv. Negro Jamapa seeds were surface-sterilized by soaking in 96% (v/v) ethanol for 30 sec followed for 7 min with 6% sodium hypochlorite solution with shaking. Seeds were subsequently washed several times with sterilized water and placed on plates containing 1% water-agar to germinate at 30°C for 48 h in the dark.

For plants grown in hydroponic conditions, seedlings of similar size were transfer to plastic trays containing 8 L of nitrogen-free Fahräeus nutrient solution (Fahraeus, 1957). After transplanting, each seedling was inoculated with 1 ml of bacterial suspension (10^8^ cells ml^–1^) of the desired bacterial strain. Nutrient solution was totally renovated 48 h later and started to be aerated with aquarium air pumps. The volume and pH (6.5) of the trays were controlled throughout the experiment. Plants were grown in a glasshouse under controlled environment conditions (25°C–27°C, 70% humidity, and natural illumination).

For nodulation kinetic, selected 2-day-old uniform seedling were transferred to growth pouches (CYG^TM^) containing 10 ml of nitrogen-free Fahräeus nutrient solution (Fahraeus, 1957) under controlled environmental conditions (14/10 h light/dark cycle, 22°C/16°C and relative humidity 60 to 70%) and were incubated 30 days. Each seedling was inoculated with 1 ml of bacterial solution (10^8^ cells ml^–1^) of the desired bacterial strain. For each bacterial strain, six growth pouches containing one seedling each were analyzed daily for nodule appearance and the number of nodules was scored each day for 30 days.

The expression analysis of selected plant and bacterial genes which participate in the rhizobial infection process, was analyzed as described by Nova-Franco et al. (2015). Briefly, 2-day-old selected uniform common bean seedlings were placed in plastic square bioassay dishes with solid nitrogen-free Fahräeus nutrient solution and were incubated in a growth chamber at 25°C, 16/8 h light/dark cycle and relative humidity 70%. Each seedling was inoculated with 1 ml of bacterial solution (10^8^ cells ml^–1^) of the desired strain. After 1 dpi, the zone of the root susceptible to rhizobium infection (hereafter referred as susceptible zone) was detached, frozen in liquid nitrogen, and stored at −80°C until used.

To evaluated the expression of the bacterial selected genes, 2-day-old selected uniform common bean seedlings were transferred into a hydroponic system and inoculated as described before. After 9 dpi, roots were detached, while at 21 dpi nodules and roots (roots from which nodules were harvested) were collected separately and frozen in liquid nitrogen, and stored at −80°C until used.

### Evaluation of Nitrogen Fixation Capacity

Nitrogenase-specific activity was determined by acetylene-reduction assay (ARA) in nodules as described by Hardy et al. (1968) using a gas chromatograph (Varian 3300). Leghaemoglobin content (Lb) was determined in plants harvested after 21, 25 and 30 dpi as previously described by LaRue and Child (1979). Fluorescence in samples was measured in a microplate reader (Synergy^TM^ H1, BioTek Instruments, Inc.) with an excitation wavelength set at 405 nm and an emission set at 650 nm. The difference in fluorescence between heated and unheated samples was proportional to heme protein content. The Lb content was determined from three biological replicates in three independent experiments.

### Nodule Histology

Collected nodules were prepared as described in Reyero-Saavedra et al. (2017). For pictures, safranine-stained semi-thin sections (25 μm) from twenty nodules of each strain detached 21 dpi were prepared using a hand-microtome and stained for 5 min with safranine in 50% ethanol before embedded in LR-White Resin were examined with a Zeiss AX10 microscope coupled to a Zeiss Axiocam 503 color digital camera (Carl Zeiss). Images were processed using ImageJ 1.52v.

### Root Hair Deformation and Rhizobia Attachment Assay

Two-day-old common bean seedling were transferred into modified nitrogen-free Fahräeus medium plates (Catoira et al., 2000) and inoculated with 1 ml suspension (O.D._600_ = 0.3) of rhizobia WT (WT/pGUS), mutant (ΔRetPC57/pGUS), or the complement (ΔRetPC57/pGUS-pc57) strains. After four and six dpi, the zone of the root susceptible to rhizobial infection (hereafter referred as susceptible zone) were collected and immersed in GUS staining solution [0.05% 5-bromo-4-chloro-3-indoxyl-β-D-glucuronic acid (X-gluc), 100 mM sodium phosphate buffer (pH 7), 0.5 mM potassium ferrocyanide, 0.5 mM potassium ferricyanide, 10 mM Na_2_EDTA and 0.1% Triton X-100] and incubated for 3 h at 37°C. To evaluate the rhizobia adhesion to the common bean roots, pictures of susceptible zones showing blue color were captured using a bright field SZX10 stereomicroscope (Olympus) equipped with an Olympus UC50 camera (Olympus). To quantify the number of rhizobia-induced root hair deformation events, susceptible zones were examined under bright-field microscopy (Velaquin) equipped with an 18 Mega-Pixels Digital Camera with Aptina CMOS Sensor (Velaquin). For these experiments, four biological replicates, each one with six roots from different seedlings, were included.

### RNA Purification and Quantitative RT-PCR Analysis

Total RNA of WT and ΔRetPC57 were purified from a culture of 60 ml (as described in Bacterial strains, plasmids, and growth conditions section). After 6 h of growing, the samples were centrifuged at 10,000 rpm for 5 min at 4°C, the pellet was stored at −80°C until used. The pellet was resuspended with 100 μl of 1% TE buffer (10 mM *Tris* – 1 mM EDTA in 1% DEPC H_2_O), then enzymatic lysis was done with lysozyme (20 mg/ml) and proteinase K (Thermo Scientific). Total RNA was isolated using an RNeasy^*R*^ Mini Kit (Qiagen) according to the manufacturer’s instructions. Total RNAs of frozen roots or nodules were purified from 250 or 100 mg, respectively, using TRIzol^TM^ Reagent (Thermo Fischer Scientific, Inc.) following manufacturer’s instructions.

RNA was quantified using a NanoDrop spectrophotometer (Thermo Fischer Scientific, Inc) and electrophoresed under denaturing conditions in agarose-formaldehyde gels. Genomic DNA was removed from RNA using DNaseI RNase-free (Thermo Fischer Scientific, Inc) in the presence of a ribonuclease inhibitor (RiboLock^TM^ RNase inhibitor, Thermo Fischer Scientific, Inc) according to the manufacturer’s instructions. Absence of genomic DNA contamination was confirmed by PCR amplification, using primers designed for the *rpoA* reference gene. To analyze the expression of selected genes, first-stranded cDNA was synthesized 2.0 mg of DNA-free total RNA by the oligo-dT (for plant genes) or the random priming (for bacterial genes) methods using Thermo Scientific RevertAid^TM^H Minus First Strand cDNA Synthesis Kit (Thermo Fisher Scientific, Inc) according to manufacturer’s instructions (Hernández et al., 2007; Ramírez et al., 2013).

The expression level of the desired genes was quantified by qRT-PCR in a CFX96^TM^ Real-Time System (Bio-Rad). The Maxima SYBR Green/ROX qPCR Master Mix (Thermo Fischer Scientific, Inc.) was used in PCR reactions using two-step cycling protocol according to the manufacturer’s instructions. The expression level of different genes was calculated with the ΔΔCt method, as reported (Hernández et al., 2007). The housekeeping genes ubiquitin *UBC9* and *rpoA* were used to normalize gene expression of plant and bacterial genes, respectively (Hernández et al., 2007; Ramírez et al., 2013). The expression of each gene was determined from three replicates in three independent samples in a single qRT-PCR experiment. Primers used in these experiments were designed by using the Oligo 7 software and are provided in Supplementary Table 4.

### Identification of *RetPC57-RetPC58* Operon

cDNA of WT strain was used as a template to obtain three PCR products. A 250 bp internal product of *RetPC57* gene was obtained using primers Up-RetPC57 and Lw-RetPC57. For *RetPC58* gene, primers Up-RetPC58 and Lw-RetPC58 were used to obtain a 250 bp internal fragment. To amplify an intergenic fragment *RetPC57*-*RetPC58* primers Up-RetPC57 and Lw-PC58gus were used to obtain a 945 bp product. The PCR reactions were carried as described in PCR amplification section. The amplification products were analyzed by agarose gel electrophoresis with ethidium bromide and capture using an image system (SmartView Pro UVC-1100 Major Science).

### Orthologs, Phylogeny, and Genomic Context Analysis

Orthologs and identity of *RHE_PC00055, RHE_PC00056, RHE_PC00057, RHE_PC00058* and *RHE_PC00059* genes were obtained from Sequence Similarity Data Base (SSDB) of KEGG (Supplementary Table 1). Protein sequences of orthologs were collected from UniProt database. A multiple sequence alignment was performed using CLUSTALW with BLOSUM as weight matrix^4^. The Interactive Tree of Life (iTOL^5^; Letunic and Bork, 2019) was used for the display and manipulation of the phylogenetic tree. Comparative genomic context analysis was performed using the Integrated Microbial Genomes (IMG^6^; Chen et al., 2019).

### Statistical Analysis

All statistical analyses were analyzed by Student’s *t*-test using the GraphPad Prism 7.0a software. Differences were statistically significant if the *P*-value was lower than 0.05 (*P* ≤ 0.05).

## Data Availability Statement

The original contributions presented in the study are included in the article/Supplementary Material, further inquiries can be directed to the corresponding author/s.

## Author Contributions

SR and LG conceived and designed the study. SR, DC-G, MR, MI-A, and MR-S performed the experiments. MS-P performed *in silico* analyses. SR, DZ-S, OV-L, GH, and LG analyzed the data and wrote the manuscript. All authors contributed to the critical revision of the manuscript, read and approved the submitted version.

## Conflict of Interest

The authors declare that the research was conducted in the absence of any commercial or financial relationships that could be construed as a potential conflict of interest.

## Footnotes

1 https://mistdb.com/genomes/GCF_000092045.1

2 https://www.kegg.jp/

3 http://www.microbesonline.org/

4 https://www.genome.jp/tools-bin/clustalw

5 https://itol.embl.de

6 https://img.jgi.doe.gov/help.html
