# Supplementary material for: A Novel OmpR-Type Response Regulator Controls Multiple Stages of the Rhizobium etli – Phaseolus vulgaris N2-Fixing Symbiosis
Source: Front Microbiol. 2020 Dec 15;11:615775. doi: 10.3389/fmicb.2020.615775 (PMC7769827; doi:10.3389/fmicb.2020.615775)
Supplement: Supplementary file 3 [file Table_3.docx]

**Table S3. Bacterial strains and plasmids used in this study.**

| **Strain or plasmid** | **Relevant characteristics** | **Reference** |
| --- | --- | --- |
| ***Rhizobium etli*** |  |  |
| CE3 | CFN42 derivative, Nal^r^, Sm^r^ | Noel et al., 1984 |
| ΔRetPC57 | CE3 Δ*RHE_PC00057*, Nal^r^, Sm^r^ | This work |
| **Plasmids** |  |  |
| pCR2.1 TOPO | Cloning vector for PCR products, Ap^r^ Km^r^ | Invitrogen |
| pJET2.1/blunt | Cloning vector for blunt ended PCR products, Amp^r^ | Thermo Fischer Scientific |
| pK**mobsacB* | Mobilizable cloning vector, Km^r^ | Schäfer et al., 1994 |
| pBBMCS53 | Δp*lacZ* pBBR1MCS-5 derivative, carrying the promoterless *uidA* gene from pWM5, Gm^r^ | Girard et al*.*, 2000 |
| pLC290 | Cumate inducible expression vector carrying the P_R/cmtO_ promoter, Km^r^ | Chubiz et al., 2013 |
| pFAJ1700 | Broad host range plasmid RK2-derived cloning vector, Tc^r^ | Dombrecht et al., 2001 |
| pRK2013 | Conjugation helper plasmid, Km^r^ | Figurski and Helinski, 1979 |
| pK::Δpc57 | pK**mobsacB* derivative containing regions flanking *RetPC57* gene, Km^r^ | This work |
| pPC57-gus | pBBMCS53 derivative *RetPC57::uidA* transcriptional fusion, Gm^r^ | This work |
| pPC56-gus | pBBMCS53 derivative *RetPC56::uidA* transcriptional fusion, Gm^r^ | This work |
| pPC58-gus | pBBMCS53 derivative *RetPC58::uidA* transcriptional fusion, Gm^r^ | This work |
| pnodTc-gus | pBBMCS53 derivative *nodTc::uidA*, Gm^r^ | This work |
| pQPC57 | pLC290 derivative carrying the *RetPC57* gene under the P_R/cmtO_ promoter, Km^r^ | This work |
| pPC57 | pFAJ1700 derivative carrying the *RetPC57* gene under its own promoter*,* Tc^r^ | This work |
| pGUS | pFAJ1700 derivative carrying the *uidA* gene under the *lacZ* promoter, Tc^r^ | Isidra-Arellano et al*.*, 2018 |
| pGUS-pc57 | pGUS derivative carrying the *RetPC57* gene under its own promoter*,* Tc^r^ | This work |
| ***Escherichia coli*** |  |  |
| DH5α | *supE*44Δ*lacU*169(φ80*lacZ*ΔM15)  hsdR17recA1endA1*gyrA*96  *thi*-1*relA*1 | Sambrook et al., 1989 |
