# Supplementary material for: A Novel OmpR-Type Response Regulator Controls Multiple Stages of the Rhizobium etli – Phaseolus vulgaris N2-Fixing Symbiosis
Source: Front Microbiol. 2020 Dec 15;11:615775. doi: 10.3389/fmicb.2020.615775 (PMC7769827; doi:10.3389/fmicb.2020.615775)
Supplement: Supplementary file 4 [file Table_4.docx]

**Table S4. Primers used in this work.**

| **Primers used for plasmid construction** | | |
| --- | --- | --- |
| **Plasmid** | **Primer/Genome position** | **Sequence 5’ → 3’** |
| pK::ΔPC57 | Up1-PC57/ pRet42c54719 | CTGAAACTTCGGCCTGCCATTCCAC |
|  | Lw1-Ov-PC57/ pRet42c55706 | GCCGGGGCATGGTCATTGCGTTGTCCGTCA |
|  | Up2-Ov-PC57/ pRet42c56391 | CGGACAACGCAATGACCATGCCCCGGCTTT |
|  | Lw2-PC57/ pRet42c57245 | CGAGAATTTCGTTGAGGAGCTCGATGATGT |
| pPC57-gus  pPC56-gus | Up-PC57gus/ pRet42c55307  Lw-PC57gus/ pRet42c56234 | GGGTCTAGACAAGAGCTGCCGCAA CAAA  CTGCTTCGATACCAACTGGCCGGCGCTG |
| pPC58-gus | Up-PC57gus/ pRet42c55307  Lw-PC58gus/ pRet42c56699 | GGGTCTAGACAAGAGCTGCCGCAA CAAA  ATA*CTCGAG*TCGGCACGGAACGGGTGATG |
| pnodTc-gus | Up-nodTcgus/ pRet42c57469  Lw-nodTcgus/ pRet42c58678 | GCGGGAT*GAGCTC*GAACGGATTTT  GCGAAGCTTATCGGGACGGTTCCGGATG |
| pQPC57 | Up-QPC57/ pRet42c55686  Lw-QPC57/ pRet42c56396 | GGATC*TCTAGA*TTCTAATGACGGACAACGCAA  GGGG*ACTAGT*GGTCAATCCAACAGCAGTTGAT |
| pPC57  pGUS-pc57 | Up-PC57-Cpl/ pRet42c55307 | AAA*ATGCAT*AGACAAGAGCTGCCGCAA |
|  | Up-PC57-Cpl/ pRet42c56392 | AAAAG*CTCGAG*GGCATGGTCAATCCAA |
|  | gusLw | ACAGGACGTAACATAAGGGACT |
| **Primers pairs used for expression analysis** | | |
| ***R. etli* CE3 genes** | | |
| **Gene name** | **Gene ID** | **Primer/ Sequence 5’ → 3’** |
| *rpoA* | *RHE_CH01699* | Forward  CCGATCGGCCTTATCCC  Reverse GTTTGCCGAACGGACCGACAG |
| *RetPC57* | *RHE_PC00057* | Forward CTGCAGGAATATCTGGTGGA  Reverse AGATTGAGGCCCGATATCCT |
| *RetPC58* | *RHE_PC00058* | Forward CAACAGAGGCTGTTCCATGA  Reverse CCGAGAATTTCGTTGAGGAG |
| *nodTc* | *RHE_PC00059* | Forward GCGTCGAAGAAGTCGAAAAC  Reverse GGCGACGTAAAGATCCACAT |
| *RetPC56* | *RHE_PC00056* | Forward GCGCACAGGTCTCTATGTCA  Reverse CCGAGGTCACTACCTTCGAC |
| *nodA* | *RHE_PD00310* | Forward CTGGAGCTTACAACGCCCTT |
|  |  | Reverse CAAACCGAACGCCAAGTTGC |
| *nodB* | *RHE_PD00282* | Forward  CACTGGTCGTCACAGCGTTT  Reverse  CGAGTATCTGACGTTGCACCT |
| *rmrA* | *RHE_PB00008* | Forward CTCGATGCCGCCGTCAAGCAA  Reverse ATCACGTAAACCGCGCTGGTC |
| *RHE_CH01192* | *RHE_CH01192* | Forward  ATGTGAAGGCCGACTACACC  Reverse  CTGTTCGATGACCGACTGCT |
| *RHE_CH03357* | *RHE_CH03357* | Forward  CTTCATCCAATTCAGCGCCG  Reverse  CCGCCTGGTTTTCATGAACG |
| *mexE1* | *RHE_CH03349* | Forward  CGGTCGGCTCAGCTCAACCTC  Reverse  TCACCATCTCTTCGCTGGCAT |
| *mexF1* | *RHE_CH03348* | Forward  GACACGCTGCTCGAAGCCAT  Reverse  CGAAAACCGCGACGAGCACC |
| *RHE_CH01305* | *RHE_CH01305* | Forward  GTCGAGGTCAGTACGGTTCC  Reverse  ACCACCACCTGTTGTTCGTT |
| *hfixL* | *RHE_PF00509* | Forward CGACGGGACGATCACGCACTG |
|  |  | Reverse TCAGCTGGTCGCGGATGT |
| *fxkR* | *RHE_PF00530* | Forward GGCAGCACCTCAGCATGAC |
|  |  | Reverse GACCGTATAACCTTC TCCTCGAA |
| *fnrNch* | *RHE_CH02479* | Forward CACGGCGGAGGAGAAGGT |
|  |  | Reverse GTGCTGATGGCGGTTTGC |
| *fnrNd* | *RHE_PD00216* | Forward GCGCGAATGGATGCTGAC |
|  |  | Reverse AGTGCTCGTCGCCGTCTC |
| *fixNd* | *RHE_PD00296* | Forward GCGCACGATCATCTGTTT |
|  |  | Reverse CGTCGCAATAGCCCGTAA |
| *nifA* | *RHE_PD00228* | Forward TATTGGCGTTCCGGTAAAAG |
|  |  | Reverse GATCGTCCGACTGACCAGAT |
| *nifH* | *RHE_PD00202* | Forward AATCGCATTTTACGGCAAAG |
|  |  | Reverse AAGGTCTTCCACCGAACCTT |
| ***P. vulgaris* genes^§^** | | |
| **Gene name** | **Gene ID** | **Primer**  **Sequence 5’ → 3’** |
| *UBC9* | *Phvul.006G110100* | Forward GCTCTCCATTTGCTCCCTGTT |
|  |  | Reverse TGAGCAATTTCAGGCACCAA |
| *CYCLOPS* | *Phvul.002G128600* | Forward TCCTTACCACATTCTGCTGAGA |
|  |  | Reverse CCAAGAGATTCCAGAGGTTCA |
| *NSP2* | *Phvul.009G122700* | Forward GACGGTTATCGGGTAGAGGAG |
|  |  | Reverse CGGAGGAAGAAGAAGTCCAAA |
| *NIN* | *Phvul.009G115800* | Forward GGGAGAAGAGGCGTACGAAG |
|  |  | Reverse GTTGTGGGACACACTCCGAT |
| *NF-YA1 (HAP2)* | *Phvul.001G196800* | Forward TACTTTGGCAATCCATCCTTG |
|  |  | Reverse AGACAGTTCGGTGCAGAAAGA |
| *ENOD40* | *Phvul.002G064200* | Forward GGGTCCTTACCCCTCACACT |
|  |  | Reverse TGTAGCCAAAGCCTCTCATCC |
| *PEPC* | *Phvul.005G066400* | Forward AAGTGAGTATGCCCCTGGTTT |
|  |  | Reverse GAAAGGGAAGATGGGTGAAAG |
| *GOGAT* | *Phvul.001G076400* | Forward ACCAGGAGGTTGTGGATTTT |
|  |  | Reverse TTTTTGCTTTCCTTCCTTCG |

The restriction sites introduced by the primers are in italic.

Underlined nucleotides indicate the overlap region among primers.

Primers for *R. etli* genes analyzed were designed using the *R. etli* genome sequence GCF_000092045.1 (González et al., 2006). Specific primers for *fnrN*ch and *nifA* genes according to Ramírez et al. (2013). Primer gusLw according Gómez-Hernández et al. (2011)

Primer sequences for *P. vulgaris* genes according to Nova et al. (2015).
